# Biofilm Formation and Plastic Degradation in Bacteria from Different Environments: Evidence for Phenotypic Acclimation and Metabolic Exaptation

**DOI:** 10.3390/microorganisms14050959

**Published:** 2026-04-24

**Authors:** Angela Conti, Debora Casagrande Pierantoni, Beatrice Strinati, Lorenzo Favaro, Laura Corte, Gianluigi Cardinali

**Affiliations:** 1 Department of Pharmaceutical Sciences, University of Perugia, 06121 Perugia, Italy; angela.conti@unipg.it (A.C.);; 2 CEMIN Excellence Research Centre, University of Perugia, 06121 Perugia, Italy; 3 Waste to Bioproducts-Lab, Department of Agronomy Food Natural Resources Animals and Environment (DAFNAE), Padova University, 35020 Padova, Italy; 4 Department of Microbiology, Stellenbosch University, Stellenbosch 7599, South Africa

**Keywords:** environmental bacteria, biofilm, plastic degradation, phenotypic acclimation

## Abstract

Microbial communities inhabiting natural and anthropogenically impacted environments are exposed to diverse abiotic stressors that can influence the distribution of functional traits. However, distinguishing the processes underlying phenotypic patterns remains challenging in microbial systems, where ecological and evolutionary dynamics often overlap. In this study, we experimentally assessed the distribution of biofilm formation and plastic degradation capacity in bacterial isolates across environments characterized by different stress regimes, to evaluate whether these traits are primarily associated with environmental context rather than phylogenetic relatedness, and may therefore reflect environment-dependent phenotypic modulation on a lineage-specific functional background. Taxonomic affiliation was assessed using 16S rRNA gene sequencing, while expressed biochemical profiles were characterized by Fourier-transform infrared (FTIR) spectroscopy. Multivariate ordination and Partial Least Squares analyses were used to explore relationships among taxonomy, biochemical profiles, functional phenotypes, and environment of isolation. Phylogenetic signal analysis confirmed that neither trait was strongly constrained by vertical inheritance, with Blomberg’s K ≈ 0 and Fritz & Purvis’ D = 0.51, consistent with environment-driven rather than phylogenetically conserved trait distributions. Both biofilm production and plastic degradation capacity showed significant environment-dependent differences in their relative frequencies (Fisher’s exact test, biofilm: *p* = 5.5 × 10^−5^; PCL degradation: *p* = 2.5 × 10^−4^) and were not directly associated with each other (Wilcoxon rank-sum test, *p* = 0.45; linear model, *p* = 0.68). Overall, these results indicate that microbial functional traits are unevenly distributed across environments and weakly constrained by taxonomy, consistent with the contribution of multiple, non-mutually exclusive processes that remain difficult to disentangle empirically.

## 1. Introduction

Microbial communities and their surrounding environments are engaged in a deeply interconnected, bidirectional relationship [1,2,3]. On one hand, microorganisms play an essential role in shaping ecosystem structure and function through nutrient cycling, signaling, and the modulation of host responses [4,5,6,7]. On the other hand, the environment produces growth and stressing conditions that exert a selective pressure on the microbial populations [8]. This equilibrium has shaped both microbial species and the environment, but is increasingly perturbed by anthropogenic pressures, that selects for traits not challenged until up the upcoming of the industrial era [9].

Understanding how microbial functional traits are distributed across environments remains a central challenge in environmental microbiology. Environmental stressors are known to influence microbial communities at multiple biological levels, yet disentangling the processes that govern the emergence and persistence of functional traits remains difficult [10,11,12]. Conceptually, these processes can be viewed as operating across at least three non-mutually exclusive levels.

At the community level, environmental conditions can act as ecological filters, favoring the persistence and proliferation of taxa whose pre-existing phenotypic characteristics are compatible with local conditions a phenomenon commonly described as habitat filtering [13,14,15,16,17]. Under this framework, community-level patterns emerge from the differential persistence and proliferation of taxa whose pre-existing phenotypic characteristics are compatible with local conditions. Increasing attention has therefore shifted from taxonomic identity alone toward the distribution of functional traits within microbial assemblages [18].

At the individual level, microorganisms may respond to environmental stressors through phenotypic plasticity or physiological acclimation [19,20,21,22], modulating the expression of functional traits without requiring changes in community structure or genetic composition. Phenotypic plasticity has been proposed as a mechanism allowing microbes to rapidly adjust to fluctuating environments, potentially influencing community assembly and functional outcomes. In this context, exposure to novel substrates, oligotrophic conditions, or sub-inhibitory concentrations of antimicrobial compounds has been associated with changes in traits such as biofilm formation, metabolic activity, and stress tolerance [23,24]. However, distinguishing induced phenotypic responses from the selective enrichment of organisms already expressing these traits remains methodologically challenging [25].

At a longer evolutionary scale, the distribution of functional traits may also reflect lineage-specific capacities shaped by past evolutionary processes. In this context, traits expressed under novel environmental conditions may arise from the co-option of pre-existing metabolic capabilities, consistent with the concept of exaptation, rather than from recent genetic innovation or strict vertical inheritance [26].

These three levels—community assembly, phenotypic plasticity, and evolutionary background—are not mutually exclusive and may operate simultaneously, generating complex patterns in the distribution of microbial functional traits across environments.

One of the most important traits in current environmental microbiology is the effect of plastics in soil and related habitats [27,28,29]. Microbial communities inhabiting natural and anthropogenically impacted environments are continuously exposed to a wide range stressors [30], among which plastic is one of the most concerning [31,32,33] both for the extension of the problem and for the nature of these xenobiotic compound [34]. These conditions are known to influence microbial survival, community composition, and functional potential, yet the processes through which microbial phenotypic traits become prevalent under such stresses remain incompletely understood [35,36,37]. Synthetic polymers represent a particularly illustrative case, as they introduce new and persistent surfaces and carbon sources into natural environments [38,39,40]. The establishment of microbial assemblages on plastic substrates, often referred to as the “plastisphere,” has been described as a dynamic process involving early colonization, succession, and functional differentiation [41]. Similar considerations apply to environments exposed to low levels of antibiotics, where microbial communities may display altered physiological states or shifts in trait distributions without overt growth inhibition [42]. In both cases, observed phenotypes may reflect a combination of environmental filtering, phenotypic plasticity, and longer-term evolutionary processes, operating simultaneously and on overlapping temporal scales.

Based on a metagenomic study, it was proposed that the addiction of plastics in soil increases the plastic-degradation functionality by increasing the abundance of degradation genes, with a concomitant reduction of the taxonomic richness [43]. Although important, these finding are limited by the lack of evidence of the effective expression and functionality of the genes. To complement metagenomic results, we analysed the actual ability to degrade plastics by strains isolated from the same substrate, i.e., litter, during the animal breeding and during maturation when various types of plastics were buried in the organic matter. The experimental characterization, through in vitro tests, of individual isolates, allowed direct measurement of expressed functional traits such as biofilm formation and plastic degradation capacity. Together with this trait we evaluated whether biofilm-forming capacity could also be affected by plastic pollution, as suggested in literature [44,45]. Our approach is intended to contribute with these empirical observations to shape future work in which metagenomics and phenotypic analyses are carried out alongside, to better interpret how environmental stresses are associated with phenotypic trait distributions in environmental microorganisms.

## 2. Materials and Methods

### 2.1. Isolation Sources

Bacterial isolates used in this study were obtained from poultry litter during intensive farming systems and during maturation when the following materials were buried for one year at a depth of 10–15 cm in the litter heap: (i) Mater-B, (ii) polylactic acid (PLA), and (iii) polyvinyl chloride (PVC). Plastic substrates consisted of entire commercial items (PLA drinking cups, Mater-B shopping bags, and medical-grade PVC infusion tubing cut into ~10 cm segments. As a control, microorganisms from other environments were isolated and analyzed for the same traits: (i) surface soil (0–5 cm depth) from Lido di Spina (Comacchio, Ferrara, Italy), characterized by elevated salinity; (ii) seed surfaces (Bavicchi S.p.A., Perugia, Italy), representing oligotrophic and water-limited conditions; (iii) wall surfaces of the Volumni Hypogeum (Perugia, Italy), characterized by oligotrophy and high relative humidity (95–100%). These environments were selected to represent conditions differing in nutrient availability, exposure to different stressors, and physicochemical constraints. This design allowed to assess whether the observed functional traits were specifically associated with litter and plastic-exposed conditions, or more generally distributed across diverse ecological contexts.

### 2.2. Bacterial Cultivation

After the sampling, plastic items were gently brushed to remove loosely attached soil particles. Microbial cells were recovered from each matrix using environment-specific protocols. Seeds and plastic samples were immersed in sterile physiological solution and agitated at 150 rpm for 30 min. Poultry litter samples were homogenized in sterile phosphate-buffered saline (PBS), while hypogeum walls were sampled using sterile swabs across multiple surface areas. Suspensions were centrifuged at 4500 rpm for 10 min, and resulting pellets were resuspended in PBS. Serial dilutions were plated on Brain Heart Infusion (BHI) agar (HiMedia Laboratories, Modautal, Germany; 1.7% agar) and incubated at 25 °C for 24–48 h. Morphologically distinct colonies were subcultured and preserved at −80 °C in BHI broth supplemented with 17% glycerol.

### 2.3. Biofilm Quantification by Crystal Violet Assay

Biofilm production was quantified using a crystal violet (CV) microtiter plate assay. Each isolate was tested in two independent biological replicates, each consisting of six technical replicates. Overnight cultures grown in BHI broth at 25 °C were adjusted to an optical density at 600 nm (OD_600_) of 0.5. Aliquots (150 μL) were transferred into 96-well polystyrene plates and incubated at 25 °C for 3 h to allow initial adhesion. Following incubation, planktonic cells were removed, and wells were washed three times with sterile PBS. Fresh BHI supplemented with 1% dextrose was added, and plates were incubated for an additional 24 h at 25 °C [46]. After washing, adherent biofilms were stained with 0.1% (*w*/*v*) aqueous crystal violet for 15 min. Excess stain was removed by washing with distilled water. Bound crystal violet was quantified by measuring absorbance at 570 nm using a TECAN Infinite 200 PRO microplate reader (Tecan Trading AG, Männedorf, Switzerland). Absorbance values were normalized relative to negative controls, and mean values were used for downstream analyses [47].

### 2.4. DNA Extraction and 16S rRNA Gene Sequencing

Genomic DNA was extracted from overnight BHI cultures using a combined chemical and mechanical lysis protocol. Briefly, cell pellets were treated with urea buffer followed by bead-beating in Triton-SDS buffer. DNA was purified by phenol–chloroform extraction and ethanol precipitation, treated with RNase A, and resuspended in molecular-grade water. The nearly full-length 16S rRNA gene was amplified using universal primers 8F and 1492R. PCR products were purified using AMPure XP beads (Beckman Coulter, Brea, CA, USA) and sequenced by Sanger sequencing (Macrogen Inc., Seoul, Republic of Korea). Sequences were quality-checked, aligned, and used for taxonomic assignment and distance-based analyses.

### 2.5. FT-IR Spectroscopy

For Fourier-transform infrared (FT-IR) spectroscopy, bacterial strains were cultured in BHI broth supplemented with 1% dextrose and harvested during exponential growth. Cell pellets were washed twice with sterile distilled water and resuspended in 210 μL of water [48]. Aliquots (35 μL) were deposited onto IR-transparent silicon microplates (Bruker, Ettlingen, Germany) and dried at 42 °C to form homogeneous films. Spectra were acquired using a TENSOR 27 FT-IR spectrometer equipped with an HTS-XT module (Bruker Optics GmbH, Ettlingen, Germany) in transmission mode over the range 4000–400 cm^−1^, at a resolution of 4 cm^−1^ with 256 scans per sample. Spectral preprocessing included quality control, baseline correction, vector normalization, and derivative computation using OPUS software (v7.0). Preprocessed spectra were used as multivariate biochemical descriptors.

### 2.6. Polycaprolactone (PCL) Degradation Assay

Polycaprolactone (PCL) degradation was assessed using BHI agar plates supplemented with an emulsified PCL substrate (Merck KGaA, Darmstadt, Germania) [49]. Plates were prepared by incorporating a PCL–acetone emulsion into molten BHI agar following established protocols [50]. Isolates were spot-inoculated and incubated at 25 °C. Degradative activity was qualitatively assessed by the presence or absence of a clear halo surrounding colonies, indicative of polymer hydrolysis. Strains were classified as PCL-degrading or non-degrading accordingly.

### 2.7. Multivariate and Statistical Analyses

All statistical analyses were performed in R (version 4.5.1). Datasets derived from biofilm assays, FT-IR spectra, and 16S rRNA sequences were aligned to include only samples present in all datasets prior to multivariate analysis.

#### 2.7.1. Principal Coordinates Analysis (PCoA)

Principal Coordinates Analysis (PCoA) was performed separately on 16S rRNA gene sequences and FT-IR spectral profiles. For 16S data, pairwise distances were calculated using a p-distance matrix on aligned sequences. This uncorrected metric was considered appropriate for the exploratory purpose of ordination, given that PCoA is used here to visualize overall patterns of sequence divergence. For FT-IR data, Euclidean distances were computed on z-score standardized spectra. PCoA ordinations were used to explore relationships between taxonomic affiliation, biochemical profiles, biofilm production, and PCL degradation capacity.

#### 2.7.2. Phylogenetic Signal Analysis

To evaluate whether the distribution of functional traits was associated with phylogenetic relatedness, phylogenetic signal was quantified for both biofilm production and plastic degradation capacity. A phylogenetic tree was reconstructed from 16S rRNA gene sequences. Sequences were aligned using the MUSCLE algorithm, and a maximum likelihood tree was inferred under a GTR+G model following initial neighbor-joining tree construction. Branch support was assessed by bootstrap resampling, and the resulting tree was midpoint rooted.

Phylogenetic signal in biofilm production (continuous trait) was assessed using Blomberg’s K and Pagel’s λ statistics, with significance evaluated by permutation tests. For plastic degradation capacity (binary trait), phylogenetic signal was evaluated using Fritz and Purvis’ D statistic. Statistical significance was assessed by comparison with null distributions generated through permutation.

All analyses were conducted in R using the packages *ape*, *phangorn*, *phytools*, and *caper*.

#### 2.7.3. Partial Least Squares Models and VIP Analysis

Partial Least Squares Regression (PLSR) was applied to evaluate the relationship between FT-IR spectra and biofilm production (continuous response variable). Model performance was assessed using cross-validation, and the optimal number of latent variables was selected based on the minimum Root Mean Squared Error of Prediction (RMSEP). Variable Importance in Projection (VIP) scores were calculated to identify spectral regions contributing to model predictions.

Partial Least Squares Discriminant Analysis (PLS-DA) was used to discriminate PCL-degrading and non-degrading isolates based on FT-IR spectra. Repeated cross-validation was applied, and model performance was evaluated using sensitivity, specificity, predictive values, and ROC curves. VIP scores were also extracted for classification models.

#### 2.7.4. Environment–Phenotype Association Analyses

The distribution of biofilm-forming and PCL-degrading isolates across environments was evaluated using contingency tables and Fisher’s exact tests. Biofilm production was classified into high and low categories based on absorbance thresholds.

Differences in biofilm production between PCL-degrading and non-degrading isolates were assessed using Wilcoxon rank-sum tests. To account for environmental effects, linear models were fitted with biofilm production as the response variable and PCL degradation capacity and environment of isolation as predictors. Model significance was evaluated by analysis of variance (ANOVA). Claude (Anthropic) was used for assistance in R code development for the phylogenetic signal analyses, including the computation of Blomberg’s K, Pagel’s λ, and Fritz & Purvis’ D statistics.

## 3. Results

### 3.1. Multivariate Ordination of Taxonomic and Metabolic Profiles

**Figure 1:**
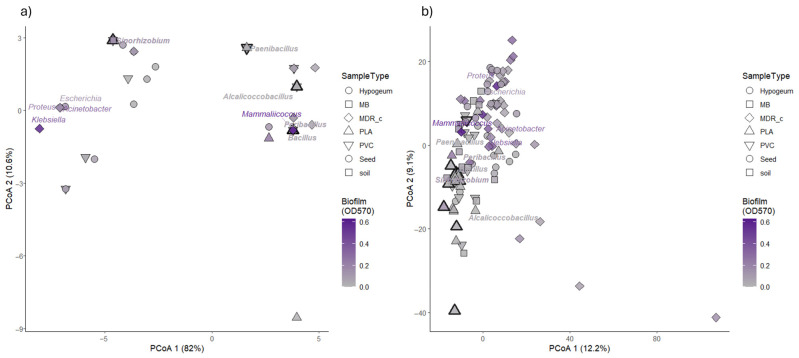
Principal Coordinates Analyses (PCoA) based on taxonomic (16S rRNA gene) and phenotypic (FTIR) signatures, overlaid with biofilm production and plastic degradation traits. (**a**) PCoA of 16S rRNA gene sequence variation among bacterial isolates, performed on p-distances calculated from aligned 16S sequences. (**b**) PCoA of FTIR spectral variation among the same isolates, performed on pairwise distances computed from scaled FTIR absorbance profiles across wavenumbers. In both panels, points represent individual isolates and are colored according to biofilm production measured as crystal violet absorbance at 570 nm (OD570), using a grey-to-purple gradient; point shapes indicate sample origin. Point outline thickness indicate PCL-degrading strains. Genus labels are displayed at centroid positions in the ordination space and colored according to the mean biofilm production of each genus. Genera containing at least one isolate able to degrade polycaprolactone (PCL) are highlighted in bold italic.

To evaluate whether the observed phenotypic traits were structured by taxonomic affiliation, we performed Principal Coordinates Analyses (PCoA) based on 16S rRNA gene sequences and FTIR spectral profiles. This multivariate approach allowed us to assess whether biofilm production and PCL degradation (Supplementary Table S1) capacity aligned with taxonomic grouping or metabolic profiling across the bacterial isolates analyzed (Figure 1).

For the taxonomic dataset, genetic distances were calculated from aligned 16S rRNA gene sequences using p-distance metric, and ordination was performed on the resulting distance matrix. The first two PCoA axes were the most informative for visualization. Although discrete genus-level clusters were not observed, isolates belonging to the same genus tended to occupy nearby regions of the ordination space, with occasional partial overlap. To visualize this tendency, genus-level centroids were calculated as the mean PCoA1 and PCoA2 coordinates of all isolates assigned to the same genus, thereby representing the average spatial position of each genus in the ordination

Biofilm production, displayed using a continuous color intensity gradient, showed a heterogeneous distribution across the PCoA space. Isolates exhibiting higher biofilm values were observed across multiple regions of the ordination rather than being confined to a single taxonomic cluster. PCL degradation capacity, indicated by increased point outline thickness, was similarly distributed across the ordination, with PCL-positive isolates occurring in different areas of the 16S-based PCoA and across multiple genera (Figure 1a).

A complementary ordination was performed using FTIR spectral data to explore similarities among isolates based on whole-cell biochemical profiles. Prior to ordination, FTIR spectra were scaled to assign equal weight to all wavenumbers, and Euclidean distances were calculated between samples. The resulting PCoA revealed a spatial distribution of isolates that but did not overlap exactly the structure observed in the 16S-based ordination

As in the PCoA distribution obtained with 16SrRNA, isolates belonging to the same genus tended to show proximity in the FTIR ordination, confirming the contribution of this technique for a taxonomic assignment, although within-genus dispersion was evident. Biofilm production values were distributed across the ordination space, with no single region exclusively associated with high or low biofilm levels. Isolates positive for PCL degradation were again detected across different regions of the FTIR ordination and were not restricted to a specific cluster or genus.

Overall, the combined ordination analyses based on 16S rRNA gene sequences and FTIR spectral profiles provide a multivariate representation of taxonomic and metabolic variability among the isolates, highlighting non-identical patterns when genetic and biochemical distances are considered. Exploratory ordination based on 16S rRNA gene sequences indicated that the major sources of variation among isolates were primarily associated with taxonomic affiliation. Phenotypic traits, including biofilm production and plastic degradation capacity, were distributed across the ordination space without clear overlapping with the main taxonomic gradients.

Consistent with these observations, quantitative analyses of phylogenetic signal revealed weak phylogenetic structuring of the measured traits. Blomberg’s K values were close to zero, indicating that closely related isolates did not exhibit more similar phenotypic values than expected under a random distribution. At the same time, Pagel’s λ values were moderate, suggesting that phylogenetic relatedness was not entirely absent but did not strongly constrain trait distribution. Similarly, the distribution of plastic degradation capacity across the phylogeny showed an intermediate pattern, with Fritz and Purvis’ D values deviating from both complete randomness and strong phylogenetic clustering.

On this basis, predictive modeling was focused on FTIR spectral data, which capture whole-cell biochemical features more directly related to expressed phenotypes.

### 3.2. FTIR-Based Prediction of Biofilm Production and Discrimination of Plastic-Degrading Isolates

As shown in the previous paragraph, there was an evident relationship between FTIR generated ordination and the ability to produce biofilm. Since this trait is particularly important for cell survival, and therefore for fitness in stressing conditions, a more accurate approach was taken to verify whether FTIR could be used to predict some of the biofilm-related characters. FTIR spectroscopy was employed in fact not as a direct proxy of functional activity, but as a high-resolution biochemical fingerprinting approach aimed at capturing intra-specific variability [51]. In this context, we tested whether spectral features could provide predictive information on biofilm formation or plastic degradation capacity. Partial Least Squares (PLS) models based on FTIR spectral data were applied to explore the relationship between whole-cell biochemical profiles vs biofilm production, measured as crystal violet absorbance at 570 nm, and polycaprolactone (PCL) degradation capacity.

**Figure 2:**
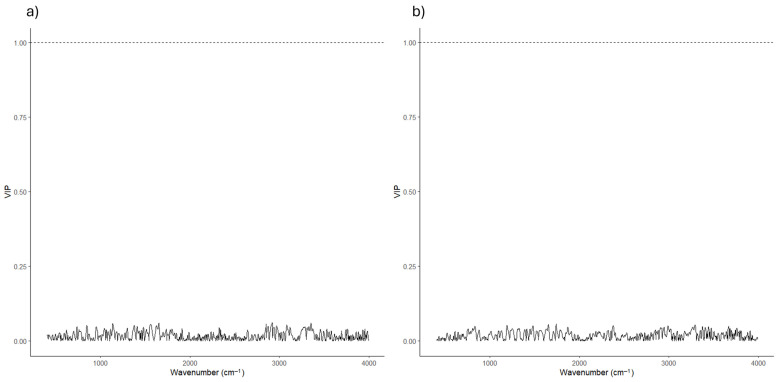
Variable importance (VIP) scores derived from FTIR-based PLS models for biofilm production and PCL degradation. Variable Importance in Projection (VIP) scores were calculated from Partial Least Squares (PLS) models built on FTIR spectral data to identify spectral regions contributing to the prediction of functional phenotypes. (**a**) VIP scores obtained from the PLS regression (PLSR) model predicting biofilm production, measured as crystal violet absorbance at 570 nm (OD_570_). (**b**) VIP scores obtained from the PLS-Discriminant Analysis (PLS-DA) model discriminating PCL-degrading (YES) and non-degrading (NO) isolates. In both panels, VIP values are plotted as a function of wavenumber (cm^−1^), and the dashed horizontal line indicates the commonly used threshold (VIP = 1) for variable relevance. No individual spectral region exceeded this threshold, suggesting that biofilm production and PCL degradation are associated with distributed biochemical patterns rather than with specific FTIR markers.

For biofilm production, a Partial Least Squares regression (PLSR) model was constructed using scaled FTIR spectra as predictors. Cross-validation was used to determine the optimal number of latent components, and model performance was evaluated based on prediction error. Overall, the PLSR model showed a weak association between FTIR spectral variation and biofilm production values (Supplementary Figure S1). Consistently, Variable Importance in Projection (VIP) scores calculated for the optimized model remained low, across the entire spectral range, with no individual wavenumber exceeding the conventional VIP threshold of 1. This pattern indicates that no specific spectral region strongly contributed to biofilm prediction and that the predictive signal was diffuse across the spectrum (Figure 2).

A complementary Partial Least Squares Discriminant Analysis (PLS-DA) was performed to classify isolates according to PCL degradation capacity (YES/NO). Model training and evaluation were conducted using repeated cross-validation with class balancing to account for the unequal distribution of degraders and non-degraders. The resulting model showed high sensitivity and negative predictive value, indicating an effective identification of non-degrading isolates (Supplementary Figure S1). In contrast, the positive predictive value remained limited. The reduced positive predictive value is consistent with the low prevalence of PCL-degrading isolates, as precision is inherently sensitive to false-positive classifications when the minority class is underrepresented.

VIP scores (Figure 2) derived from the PLS-DA model similarly showed low to moderate values across the FTIR spectrum, with no single wavenumber displaying a dominant contribution to class discrimination. As observed for the biofilm model, the discriminatory information appeared to be distributed across multiple spectral regions rather than concentrated in specific peaks.

Together, these analyses indicate that FTIR-based multivariate models captured limited associations with the phenotypic traits examined, with distributed spectral contributions and no clearly dominant biochemical signatures emerging from the VIP-based feature ranking.

### 3.3. Environment-Dependent Distribution of Functional Phenotypes

Considering that the isolates were obtained from litter at different time of maturation, the distribution of phenotypical traits among isolates is expected to reflect the different conditions more than the mere impact of high-level organic matter available in the isolation substrate. The isolations were therefore planned from very similar substrates with the major difference that one set was obtained from a chicken farm with animals present over the litter and no plastic materials. On the contrary, the other isolation set was derived from plastic items buried in litters in absence of animals. In this way, the presence of animals and the presence of plastic items are the two significant environmental variables of the whole experiment.

**Figure 3:**
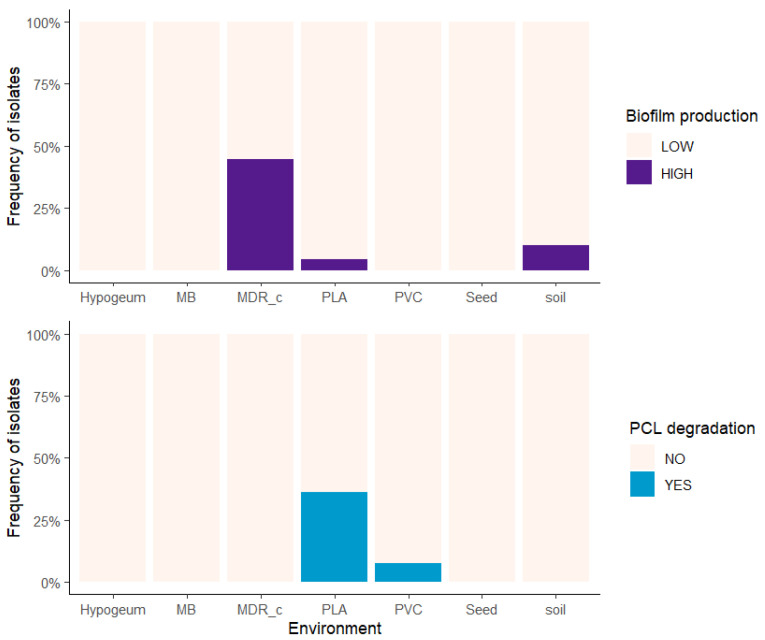
Environment-dependent distribution of biofilm production and PCL degradation phenotypes. Stacked bar plots show the relative frequency (%) of bacterial isolates (y-axis) across different environments of isolation (x-axis), classified as high (HIGH) or low (LOW) biofilm producers (top panel), and as PCL degraders (YES) or non-degraders (NO) (bottom panel). Biofilm classes were defined using a threshold of OD_570_ > 0.15. Environments include poultry litter (MB: Mater-B; MDR_c: broiler litter; PLA: polylactic acid plastics; PVC: polyvinyl chloride plastics), as well as control environments (Hypogeum: hypogeal wall surfaces; Seed: seed surfaces; Soil: standard soil).

The distribution of functional phenotypes among bacterial isolates was examined across environments of isolation to assess whether biofilm production and plastic degradation capacity varied in relation to environmental context (Figure 3).

The relative frequency of high and low biofilm-forming isolates differed significantly among environments. When isolates were classified into high and low biofilm producers based on crystal violet absorbance, their proportions varied across environmental categories (Fisher’s exact test, *p* = 5.5 × 10^−5^, bias-corrected Cramér’s V = 0.47, 95% CI [0.14, 0.64]). Similarly, the proportion of isolates capable of polycaprolactone (PCL) degradation was not uniform across environments, with significant differences in frequency observed among isolation sources (Fisher’s exact test, *p* = 2.5 × 10^−4^, bias-corrected Cramér’s V = 0.46, 95% CI [0.12, 0.63]). These patterns indicate a significant association between environment of isolation and the distribution of both functional phenotypes.

**Figure 4:**
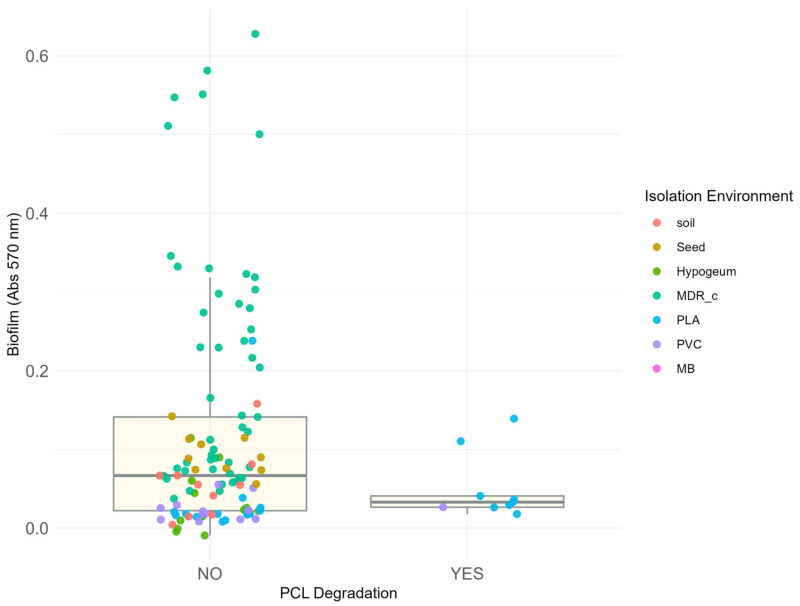
Relationship between biofilm production and the ability to degrade polycaprolactone (PCL) in isolated bacterial strains. The figure shows the absorbance at 570 nm (y-axis) as a function of the ability to degrade PCL (x-axis), classified into two categories: “NO” for strains in which no degradation halo was observed and “YES” for strains around which a degradation halo formed. Each point corresponds to a bacterial isolate, coloured according to the isolation environment. Only 9 strains showed this ability, all coming exclusively from polymer matrices (PLA—blue and PVC—lilac) previously buried in the soil. It should be noted that the latter are characterised by low absorbance values at 570 nm, indicating a poor correlation between biofilm production and degradative capacity. Conversely, all highly biofilm-producing strains are classified in the area of the graph indicating no degradation (points corresponding to “NO”). The box plots indicate the average absorbance and statistical distribution of the two categories.

To further evaluate the relationship between biofilm production and PCL degradation capacity, biofilm levels were compared between PCL-degrading and non-degrading isolates (Figure 4).

Biofilm production did not differ significantly between the two groups (Wilcoxon rank-sum test, *p* = 0.45, r = 0.07, 95% CI [0.003, 0.21]), and the distributions of biofilm values largely overlapped. Biofilm production was also modelled using a multiple linear regression framework including PCL degradation capacity and environment of isolation as predictors. When biofilm production was introduced in a model as a function of PCL degradation capacity and environment of isolation, PCL degradation was not a significant predictor of biofilm levels (linear model, *p* = 0.68, ω^2^ = 0.01). In contrast, environment of isolation showed a strong and significant association with biofilm production (*p* < 10^−6^, ω^2^ = 0.30, 95% CI [0.15, 1.00]; R^2^ = 0.35). Among individual environments, MDR_c was the only significant predictor (β = 0.157, *p* < 0.001), consistent with the hypothesis that biofilm formation is enriched under the selective pressure of antimicrobial compounds present in intensive farming conditions

Together, these analyses indicate that while both biofilm production and PCL degradation capacity are unevenly distributed across environments, the two phenotypes are not directly associated with each other. Instead, variation in biofilm production is more strongly associated with the environment of isolation than with plastic degradation capacity.

## 4. Discussion

Understanding how microbial functional traits are distributed across environments remains a central challenge in environmental microbiology. While metagenomics has given a relevant push in understanding the dynamics of traits, it cannot explain how these traits are organized in the actual microorganisms. In this study, we examined the distribution of two functional phenotypes, biofilm production and plastic degradation capacity, across bacterial isolates obtained from the same substrate (litter) present in two different environments characterized by diverse stress regimes. Plastic degradation was the primary goal of this paper, while biofilm was studied because some authors suggested that it can improve the degradation of plastic items, by anchoring microbes on their surfaces [52,53].

One of the clearest patterns emerging from our analyses is the little, if any, correspondence between taxonomic relatedness, as inferred from 16S rRNA gene sequences, and the distribution of functional phenotypes, in fact both biofilm production and plastic degradation capacity were distributed across this taxonomic space, without clear alignment to the taxonomic distribution. To further explore whether the observed distribution of functional traits reflected phylogenetic constraints, we quantified phylogenetic signal using Blomberg’s K and Pagel’s λ on biofilm and Fritz & Purvis’ D on plastic degradation ability. Biofilm forming capacity showed weak phylogenetic signal (K ≈ 0), indicating that closely related isolates did not exhibit more similar phenotypic values than expected under a random distribution. At the same time, Pagel’s λ and Fritz & Purvis’ D values were moderate, suggesting that phylogenetic structure is not entirely absent, but rather provides a background within which phenotypic variation is expressed. It should be noted, however, that the moderate values of Pagel’s λ and Fritz & Purvis’ D may partly reflect a confounding effect between phylogenetic structure and sampling design: if habitat filtering preferentially selects phylogenetically related taxa in each environment—as suggested by the uneven taxonomic distribution observed across isolation sources—then the detected phylogenetic signal may be an artefact of community composition rather than evidence of trait conservatism per se. This is because both Pagel’s λ and Fritz & Purvis’ D quantify how trait variation is distributed across a phylogenetic tree, and are therefore sensitive to the taxonomic composition of the sampled community. If habitat filtering has preferentially enriched phylogenetically cohesive taxa in each environment, isolates sharing both a trait and an environment will tend to be phylogenetically related not because the trait is inherited, but because the community itself is phylogenetically structured. Under this scenario, moderate λ and D values would reflect the phylogenetic non-randomness of the sampled community rather than true conservation of the functional trait across the tree. Together, these results suggest that broad phylogenetic lineages differ in their capacity to express certain functional traits, reflecting deep evolutionary constraints on metabolic repertoires, while fine-scale phenotypic variation within those lineages is largely driven by environmental context. This observation is consistent with growing evidence that taxonomic markers, while informative for phylogenetic placement, often provide limited insight into phenotypic and functional variability [54,55], suggesting that metabarcoding and metagenomics should be evaluated in parallel.

Similarly, FTIR fingerprinting, that proved to carry a significant taxonomic signal, had no ability to provide descriptors somehow related to biofilm production and plastic degradation. FTIR spectra capture global biochemical composition rather than specific metabolic pathways, potentially limiting their ability to resolve complex functional traits. In addition, phenotypes such as biofilm formation and plastic degradation are highly variable and context-dependent, which may reduce the consistency of associated spectral features. Finally, the combination of high-dimensional spectral data and a relatively limited sample size may further constrain model performance. Furthermore, FTIR fingerprinting, as applied in this study, primarily captured biochemical variation associated with taxonomic structure, rather than functional specialization. The lack of predictive power for biofilm formation and plastic degradation is therefore consistent with the results observed with 16S Sequencing, suggesting that global biochemical profiles do not directly reflect the expression of specific ecological functions

These results confirm the decoupling between taxonomy and the distribution of functional traits which are rather associated with environmental contexts [56].

In contrast to the weak association with taxonomy, both functional phenotypes examined in this study displayed strong environment-dependent patterns. Considering that the substrate was substantially the same, it is important to stress that for this experiments the difference across environments is primarily due to the presence of growing boilers in one case and of plastics items in the other. The relative frequencies of high and low biofilm-forming isolates, as well as the proportion of plastic-degrading isolates, differed significantly across environments of isolation suggesting that biofilm was instrumental for survival in intensive farming conditions and plastic degradation gave a nutritional advantage to species carrying plastic-degrading genes. Whereas the second case is in line with the expectations [57,58], the presence of biofilm during the breeding could be due to harsher conditions, including the presence of disinfectants and antibiotics, that favour the presence of biofilm-forming bacteria, able to use this trait to improve the rate of survival [59,60,61,62]. Furthermore, this observation leads to the evidence that the presence of animals subject to antibiotics triggered the presence of bacteria with several antibiotic resistances and ability to form biofilm. Similarly, plastic items were associated with the presence of bacteria able to degrade these polymers to some extent [43]. In systems exposed to biodegradable plastics, the release of low–molecular weight carbon compounds during polymer hydrolysis may locally increase carbon availability, potentially altering microbial metabolic activity and community composition, as reported in soil plastisphere studies [63,64,65]. In these contexts, biodegradable polymers are not ecologically inert substrates but may represent localized carbon inputs that render visible pre-existing enzymatic capacities in taxa already equipped to process structurally complex organic molecules—a mechanism more consistent with exaptation than with de novo trait evolution or classical habitat filtering. In fact, microorganisms possessing broader metabolic repertoires or pre-existing capacities to process complex substrates may be more likely to express these traits under carbon-enriched conditions. Our results are consistent with this framework, as PLA-associated isolates included taxa known for metabolic versatility and extracellular enzymatic activity. Notably, the PLA-associated degradative isolates recovered in this study included *Bacillus velezensis*, *Bacillus mojavensis*, *Bacillus inaquosorum*, *Alcalicoccobacillus plakortidis*, *Peribacillus frigoritolerans*, and *Sinorhizobium meliloti*. Members of Bacillaceae are widely documented producers of extracellular hydrolases, including esterases and lipases, which are mechanistically consistent with the hydrolysis of polyester-based polymers such as PLA. Similarly, *Sinorhizobium meliloti*, although primarily known as a plant-associated Alphaproteobacterium, possesses a relatively large and flexible genome supporting broad metabolic capabilities. The recovery of these taxa from PLA-exposed environments, together with experimentally confirmed degradative activity, suggests that polymer-associated carbon inputs may preferentially favour microorganisms with pre-existing enzymatic and metabolic breadth.

Rather than indicating the emergence of novel degradative capacities, these findings are consistent with the activation or ecological prominence of lineages already predisposed to exploit heterogeneous organic substrates. This pattern is consistent with exaptation—whereby traits evolved in other functional contexts, such as the hydrolysis of complex natural substrates, are co-opted under novel selective conditions without requiring de novo genetic innovation.

While classical ecological theory often frames environmental effects primarly in terms of selection, acting on genetic variation, microbial systems can respond to environmental conditions through multiple, hierarchically nested mechanisms operating across different temporal and biological scales. At the community level, habitat filtering reshapes assemblage composition by differentially retaining taxa whose pre-existing traits are compatible with local conditions. At the individual level, phenotypic plasticity and acclimation could allow single organisms to modulate trait expression in response to environmental signals, without alteration of the underlying genome. At the evolutionary level, exaptation may enable the co-option of pre-existing molecular capacities for novel functions under new selective contexts. These mechanisms are not mutually exclusive and may operate simultaneously, generating environment-associated phenotypic patterns that are difficult to attribute to any single process—as the results of the present study illustrate.

In this context, environments may not only select for microorganisms already expressing advantageous traits but also contribute to the stabilization or reinforcement of certain phenotypic states through repeated exposure. Such “acclimation” may reflect the stabilization of phenotypic responses under repeated environmental exposure [66].

The lack of a direct association between biofilm production and plastic degradation capacity in our dataset further supports the idea that functional traits may respond independently to environmental conditions. Although both traits were unevenly distributed across environments, they were not correlated with each other, suggesting that environmental pressures do not act uniformly across functional dimensions. Instead, environments may shape a mosaic of phenotypic outcomes, reflecting multiple, potentially overlapping responses rather than a single adaptive trajectory.

Our findings align with the perspective that ecological and evolutionary processes in microbial communities are deeply intertwined and difficult to separate empirically [25]. Environmental filtering, phenotypic plasticity, exaptation, and longer-term evolutionary change may all contribute to shaping functional trait distributions, often simultaneously. Rather than resolving this distinction, the present study highlights the value of an integrative approach that combines taxonomic, biochemical, and phenotypic data to characterize microbial diversity. Together, these approaches offer a more nuanced view of how microbial functional traits are distributed across environments subject to different stress regimes.

## 5. Conclusions

This study examined the distribution of biofilm production and plastic degradation capacity in bacterial isolates obtained from environments characterized by different abiotic and anthropogenic stressors. Our results showed that both functional traits have no correspondence to taxonomic affiliation but were associated with specific environmental conditions where their presence gave and advantage. Biofilm probably helped in harsh conditions with various biocides, whereas plastic gave an additional nutrient to those bacteria able to degrade it. By focusing on isolated strains from environmentally distinct contexts, this study provides empirical evidence that functional phenotypes are unevenly distributed across environments and weakly constrained by taxonomic affiliation.

Data suggested that environment-associated phenotypic patterns reflect two non-mutually exclusive processes: (i) phenotypic acclimation, whereby prolonged exposure to specific environmental stressors stabilizes distinct phenotypic states in organisms sharing the same genetic background; and (ii) exaptation, whereby pre-existing metabolic capacities—phylogenetically structured among broad taxonomic lineages—are co-opted under novel selective contexts such as biodegradable polymer exposure. These observations reinforce the need for caution when inferring functional potential from taxonomic composition alone and emphasize the importance of considering environmental context when interpreting microbial phenotypes.

## Data Availability

The original DNA sequences data presented in the study are openly available in NCBI GenBank at SUB16137108.

## Supplementary Materials

The following supporting information can be downloaded at: https://www.mdpi.com/article/10.3390/microorganisms14050959/s1, Figure S1: Limited predictive performance of FTIR-based PLSR model for biofilm production and PCL degradation capacity; Table S1: Phenotypic characteristics of bacterial isolates in terms of biofilm production and PCL degradation capacity.

## Abbreviations

The following abbreviations are used in this manuscript:

PCL	Polycaprolactone
PLSR	Partial Least Squares Regression
PCoA	Principal Coordinates Analysis
