# Supplementary material for: Biofilm Formation and Plastic Degradation in Bacteria from Different Environments: Evidence for Phenotypic Acclimation and Metabolic Exaptation
Source: Microorganisms. 2026 Apr 24;14(5):959. doi: 10.3390/microorganisms14050959 (PMC13209391; doi:10.3390/microorganisms14050959)

Supplementary Table S1. Phenotypic characteristics of bacterial isolates in terms of biofilm production and PCL degradation capacity

List of bacterial isolates included in this study, reporting taxonomic assignment (Genus and Species), quantitative biofilm production expressed as OD_{570} values, and qualitative PCL degradation capacity (YES/NO) based on halo formation assay

Sample_type	Species	Abs570-biofilm	Degradation capacity
Hypogeum	Paenibacillus tylopili	0.044691668	NO
Hypogeum	Tsukamurella hongkongensis	0.021925	NO
Hypogeum	Pseudomonas segetis	0.060375002	NO
Hypogeum	Streptomyces venezuelae	0.009141666	NO
Hypogeum	Streptomyces venezuelae	0.004291667	NO
Hypogeum	Microbacterium maritypicum	0.009891667	NO
Hypogeum	Streptomyces venezuelae	0.026008335	NO
Hypogeum	Agromyces cerinus subsp. nitratus	0.023375	NO
Hypogeum	Streptomyces cremeus	0.01525	NO
Hypogeum	Paeniglutamicibacter kerguelensis	0.000641668	NO
Hypogeum	Nocardia fluminea	0.089975	NO
Hypogeum	Virgibacillus halotolerans	0.114658335	NO
MDR_c	Proteus mirabilis	0.252533336	NO
MDR_c	Kurthia	0.083591665	NO
MDR_c	Kurthia	0.093025002	NO
MDR_c	Staphylococcus saprophyticus	0.165558334	NO
MDR_c	Kurthia	0.547108329	NO
MDR_c	Escherichia coli	0.229825002	NO
MDR_c	Kurthia	0.074933333	NO
MDR_c	Proteus mirabilis	0.273833331	NO
MDR_c	Escherichia coli	0.237891667	NO
MDR_c	Staphylococcus arlettae	0.047366668	NO
MDR_c	Klebsiella pneumoniae	0.279441671	NO
MDR_c	Kurthia	0.066466666	NO
PVC	Brucella intermedia	0.055341666	NO
PVC	Priestia aryabhattai	0.051133334	NO
PVC	Pseudomonas aeruginosa	0.01135	NO
PVC	Achromobacter spanius	0.008691667	NO
PVC	Pseudomonas aeruginosa	0.029533331	NO
PVC	Bacillus pseudomycoides	0.022658334	NO
PVC	Bacillus pumilus	0.018474999	NO
PVC	Alcalicoccobacillus plakortidis	0.018433333	NO
PVC	Paenibacillus xylanilyticus	0.026741667	YES
PVC	Bacillus pseudomycoides	0.021558332	NO
PVC	Pseudomonas koreensis	0.025566667	NO
PVC	Peribacillus asahii	0.011766667	NO
PVC	Lysinibacillus pakistanensis	0.010958332	NO
MDR_c	Staphylococcus piscifermentans	0.112408332	NO
MDR_c	Escherichia coli	0.062875	NO

MDR_c	Proteus mirabilis	0.329933332	NO
MDR_c	Klebsiella pneumoniae	0.500325004	NO
MDR_c	Aerococcus viridans	0.055733335	NO
MDR_c	Acinetobacter guillouiae	0.345716669	NO
MDR_c	Klebsiella pneumoniae	0.581116666	NO
MDR_c	Kurthia gibsonii	0.063766666	NO
MDR_c	Kurthia gibsonii	0.297833333	NO
MDR_c	Staphylococcus	0.063966667	NO
MDR_c	Enterococcus	0.047499999	NO
MDR_c	Staphylococcus carnosus	0.143141668	NO
PLA	Sinorhizobium meliloti	0.110608333	YES
PLA	Bacillus inaquosorum	0.018033332	YES
MB	Priestia aryabhattai	0.019075001	NO
MDR_c	Proteus mirabilis	0.229470837	NO
MDR_c	Kurthia	0.0836125	NO
MDR_c	Kurthia	0.09997917	NO
MDR_c	Escherichia coli	0.072845831	NO
MDR_c	Proteus mirabilis	0.285054173	NO
MDR_c	Klebsiella pneumoniae	0.322799997	NO
MDR_c	Proteus mirabilis	0.204108332	NO
MDR_c	Staphylococcus arlettae	0.019858333	NO
MDR_c	Staphylococcus gallinarum	0.069541665	NO
PLA	Alcalicoccobacillus plakortidis	0.00835	NO
PLA	Priestia aryabhattai	0.022858335	NO
PLA	Priestia megaterium	0.018383333	NO
PLA	Priestia aryabhattai	0.01645	NO
PLA	Priestia aryabhattai	0.018275001	NO
PLA	Bacillus velezensis	0.139150001	YES
PLA	Paenibacillus cineris	0.020791667	NO
PLA	Bacillus haynesii	0.238024997	NO
PLA	Alcalicoccobacillus plakortidis	0.029349999	YES
PLA	Bacillus velezensis	0.036416667	YES
PLA	Bacillus mojavensis	0.026516666	YES
PLA	Peribacillus frigoritolerans	0.038675	NO
PLA	Peribacillus frigoritolerans	0.033183335	YES
PLA	Alcalicoccobacillus plakortidis	0.041075001	YES
PLA	Alcalicoccobacillus plakortidis	0.022333333	NO
PLA	Priestia aryabhattai	0.026208333	NO
PLA	Bacillus pseudomycoides	0.017325	NO
PLA	Bacillus pacificus	0.018108333	NO
PLA	Bacillus pacificus	0.010108334	NO
PLA	Bacillus mycoides	0.01455	NO
soil	Peribacillus frigoritolerans	0.014899999	NO
soil	Priestia aryabhattai	0.055016667	NO
soil	Brucella anthropi	0.158191665	NO
soil	Bacillus sp.	0.081175	NO
soil	Peribacillus frigoritolerans	0.067066669	NO

soil	Rossellomorea aquimaris	0.066883334	NO
soil	Bacillus pseudomycoides	0.017275001	NO
soil	Priestia aryabhattai	0.004575	NO
soil	Metabacillus idriensis	0.055391668	NO
soil	Bacillus pseudomycoides	0.041475001	NO
Seed	Bacillus licheniformis	0.090083334	NO
Seed	Pantoea agglomerans	0.074391667	NO
Seed	Curtobacterium plantarum	0.142175001	NO
Seed	Peribacillus frigoritolerans	0.114858333	NO
Seed	Pseudomonas azotoformans	0.056125002	NO
Seed	Pseudomonas kribbensis	0.106549998	NO
Seed	Pseudomonas glycinae	0.073874999	NO
Seed	Pseudomonas boreofloridensis	0.076199999	NO
Seed	Peribacillus frigoritolerans	0.113316664	NO
Seed	Enterobacter asburiae	0.088491666	NO
MDR_c	Klebsiella pneumoniae	0.318766665	NO
MDR_c	Kurthia	0.058175	NO
MDR_c	Glutamicibacter soli	0.122616668	NO
MDR_c	Corynebacterium glutamicum	0.141333333	NO
MDR_c	Kurthia	0.077708333	NO
MDR_c	Klebsiella pneumoniae	0.303020836	NO

Figure S1. Limited predictive performance of FTIR-based PLSR model for biofilm production and PCL degradation capacity.

a) Scatter plot of observed versus predicted biofilm production values (OD_{570}). The dashed line represents the 1:1 relationship. The dispersion of points indicates a weak association between predicted and observed values.
b) Receiver operating characteristic (ROC) curve derived from PLS-Discriminant Analysis (PLS-DA) model. The deviation of the curve from the diagonal reflects limited discriminative ability of the model.

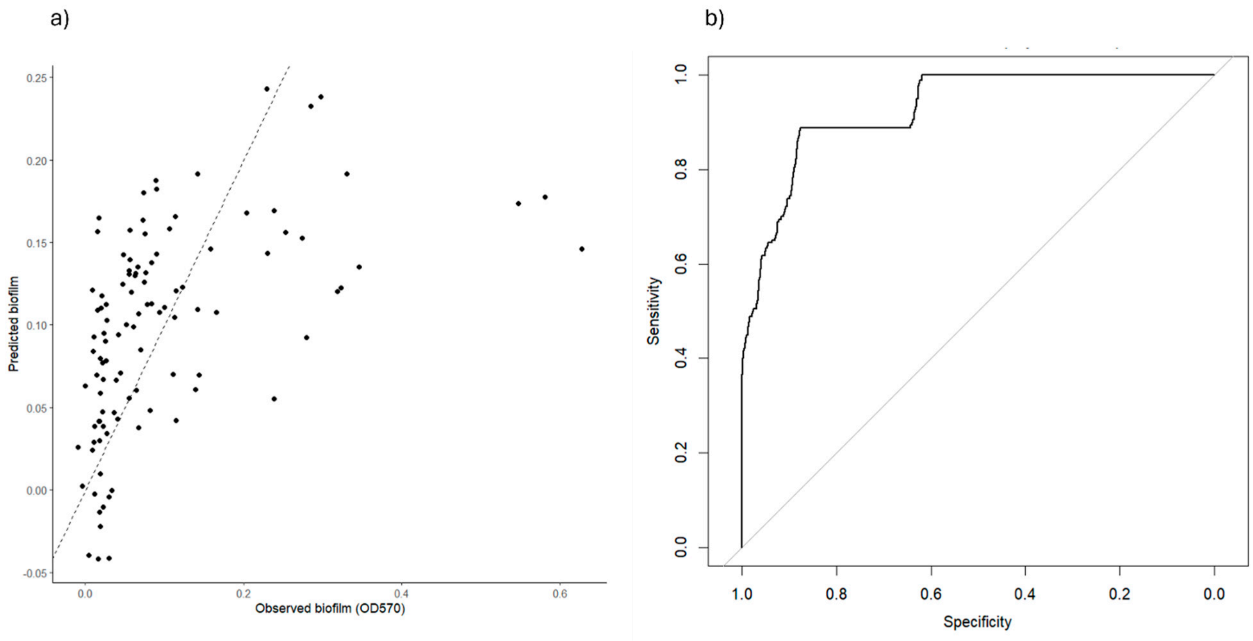

Supplement: Supplementary file 1 [file microorganisms-14-00959-s001.zip › microorganisms-4248058-supplementary.pdf]
